# Using a Genetic Algorithm as an Optimal Band Selector in the Mid and Thermal Infrared (2.5–14 μm) to Discriminate Vegetation Species

**DOI:** 10.3390/s120708755

**Published:** 2012-06-27

**Authors:** Saleem Ullah, Thomas A. Groen, Martin Schlerf, Andrew K. Skidmore, Willem Nieuwenhuis, Chaichoke Vaiphasa

**Affiliations:** 1 Faculty of Geo-Information Science and Earth Observation (ITC), University of Twente, P.O. Box 217, 7500 AE Enschede, The Netherlands; E-Mails: groen@itc.nl (T.A.G.); skidmore@itc.nl (A.K.S.); nieuwenhuis@itc.nl (W.N.); 2 Centre de Recherche Public-Gabriel Lippmann (CRPGL), L-4422 Belvaux, Luxembourg; E-Mail: schlerf@lippmann.lu; 3 Department of Survey Engineering, Faculty of Engineer, Chulalongkorn University, 10330 Bangkok, Thailand; E-Mail: vaiphasa@alumni.itc.nl

**Keywords:** genetic algorithms, thermal infrared remote sensing, spectral separability, spectral emissivity

## Abstract

Genetic variation between various plant species determines differences in their physio-chemical makeup and ultimately in their hyperspectral emissivity signatures. The hyperspectral emissivity signatures, on the one hand, account for the subtle physio-chemical changes in the vegetation, but on the other hand, highlight the problem of high dimensionality. The aim of this paper is to investigate the performance of genetic algorithms coupled with the spectral angle mapper (SAM) to identify a meaningful subset of wavebands sensitive enough to discriminate thirteen broadleaved vegetation species from the laboratory measured hyperspectral emissivities. The performance was evaluated using an overall classification accuracy and Jeffries Matusita distance. For the multiple plant species, the targeted bands based on genetic algorithms resulted in a high overall classification accuracy (90%). Concentrating on the pairwise comparison results, the selected wavebands based on genetic algorithms resulted in higher Jeffries Matusita (J-M) distances than randomly selected wavebands did. This study concludes that targeted wavebands from leaf emissivity spectra are able to discriminate vegetation species.

## 1. Introduction

Hyperspectral sensors, because of their high spectral detail over contiguous narrow bands, have proven to be a valuable tool for discriminating plants species [1–4] compared to multispectral resolution sensors [5]. However, due to high dimensionality, working with hyperspectral data poses challenging problems such as redundancy, intensive computation, and singularity of covariance matrix inversion [6–10]. To overcome these problems, the dimensionality of hyperspectral data needs to be reduced without compromising the information content. The dimensionality of the data is reduced through either band extraction or band selection [6]. In band selection a subset of the original bands is selected without affecting the physical meaning of the selected bands. In band extraction a certain number of bands is selected after transforming the original dataset [11]. Band selection is often preferred to band extraction as the physical meaning of the data remains unchanged [6,12–15].

Genetic algorithms constitute problem solving optimization methods based on the philosophy of genetics and natural selection through “survival of the fittest” [16,17]. A genetic algorithm is a popular band selector and dimensionality reduction procedure for spectral analysis [8,18–22]. The genetic algorithm as a band selector has performed with higher accuracy than other band selection algorithms for both synthetic [23] and real remote sensing data [8,18,19,24]. In remote sensing, genetic algorithms selected spectral bands for classification with hyperspectral data, as well as bands sensitive to the chemical content of plants and soils [18,19]. The majority of the studies used genetic algorithms as a band selector where the class information was broad (*i.e.*, the spectral signatures of the different classes were distinct from each other) [25] and the genetic algorithms easily selected bands that differentiated between various classes. Using visible to short-wave infrared (VIS–SWIR; 0.4–2.5 μm) spectra, Vaiphasa *et al.* [8] discriminated between sixteen mangrove plant species with similar spectral characteristics. The present study extends the genetic algorithms to the mid to thermal infrared for optimal band selection for discriminating plant species.

Till recently, vegetation spectra in the mid to thermal infrared (2.5–14 μm) was perceived as a line without any spectral features [26]. However, the introduction of spectroradiometers sensitive to mid and thermal infrared revealed that certain spectral features are associated with the composition of leaf epidermal materials (*i.e.*, cell walls and cuticular membranes), which can act as a fingerprint for discriminating vegetation [26–29]. The present study attempts to discriminate between 13 broadleaf vegetation species using genetic algorithms from high resolution mid to thermal infrared data (2.5–14.0 μm, comprising 3,024 spectral bands). The possibility of using genetic algorithm-based selected features for distinguishing vegetation species (from laboratory measured emissivity spectra) will be an important prerequisite for adjusting band positions of air-borne and space-borne floristic mapping campaigns.

## 2. Materials and Methods

### 2.1. Leaf Sampling

The dataset of leaf samples used in this study was the same as used in the [29]. The leaves were collected (between July and September 2010) from thirteen plant species (Table 1) species. To avoid pseudo-replication, leaves were collected from at least ten different plants of the same species. Leaves were acquired from different part of the plant (both on the sun and the shaded side). The leaves, attached to small twigs, were brought to the laboratory within 5 minutes, and placed in moist cotton to avoid desiccation. Spectral measurements were recorded as soon as possible.

### 2.2. Spectral Measurements

**Figure 1.**
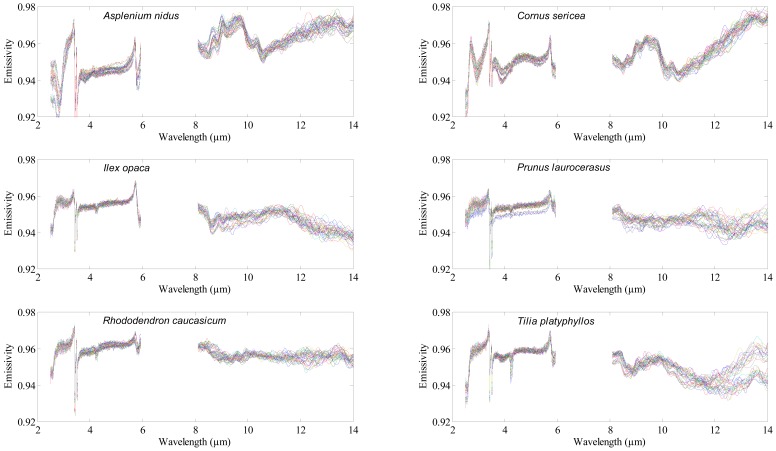
The spectral emissivity profiles of the six plant species in the mid-wave and thermal infrared domain.

A Bruker VERTEX 70 FTIR spectrometer (Bruker Optics GmbH, Ettlingen, Germany) was used to acquire the Directional Hemispherical Reflectance (DHR) spectrum of each leaf. Nitrogen (N_2_) gas was used to continuously purge the spectrometer from water vapor and carbon dioxide. A mid-band mercury-cadmium-tellurium (MCT) detector cooled with liquid nitrogen was used to measure the DHR spectrum of the adaxial (upper) surface of the leaf samples between 2.5 and 14 μm (Figure 1), with a spectral resolution of 4 cm^−1^. Thirty five (35) leaves were measured per species, thus 455 leaves were measured in total. Each leaf measurement was referenced against a calibration measurement of gold plate (infragold; Labsphere reflectance technology) with a high reflectance (approximately 96%). One thousand (1,000) scans were averaged to produce each leaf spectrum. The spectra between 6 to 8 μm were noisy (due to water absorption) and were excluded from the analysis. The DHR spectra were converted to emissivity using Kirchhoff's law (Emissivity = 1 − *R*) [30–32]. For further detail about the spectrometer and data acquisition, see [29,33].

### 2.3. Concept of Genetic Algorithm

Genetic algorithms, introduced for the first time by Holland [17], are a popular type of evolutionary optimization computation based on the concept of natural selection. The innovation behind genetic algorithms is the random (stochastic) model that uses a population of solutions rather than a single solution. During each iteration, solutions are represented in the form of a “chromosome”, with selected wavelength bands positioned as “genes”. The algorithm commences with a population of random solutions, termed the first generation. A fraction of these solutions, with the best “fitness” according to a pre-defined objective function are then selected to produce (*i.e.*, undergo the mechanism of crossover and mutation) a second generation that consists of hybridized offspring of the first generation. Of this second generation, again the solutions with the highest fitness are selected to reproduce a third generation, and so on, until the improvement in fitness between subsequent generations levels off to a pre-set threshold. Parameters that have to be selected before starting the algorithm are the chromosome size (*i.e.*, how many bands can be selected per solution), the population size (*i.e.*, the number of solutions per generation), the fraction of a generation that is selected to be the “parents” for the new generation, and when to stop the algorithm. The reproduction operators, objective function, and selection mechanism are summarized in the next subsection, while the detailed practical implementation (step by step procedure) can be found in Goldberg [16]. The genetic algorithms script was written at the Faculty of Geo-Information Science and Earth Observation (ITC), the Netherlands.

#### 2.3.1. Reproduction Operators

For problem solving, the selected chromosomes directly undergo crossover and mutation. In the crossover operation the two selected parent chromosomes merge and produce offspring (new chromosomes) that share the properties of both parents. A single point crossover was used in this study, where two parent chromosomes split into four segments (two segments per parent). Then the exchange of gene segments produces two offspring from every two parents. In mutation, a single gene (band, in this case) in the offspring chromosome is randomly altered and as a result the characteristics of the offspring differ from the parental chromosome combination.

#### 2.3.2. Objective Function

An objective function is required to assign a value to each chromosome. The associated value of each chromosome is an indication how well it fits the solution it represents. The spectral angle mapper (SAM) nearest neighbour classifier was used to evaluate the fitness values (in this case the overall classification accuracy) of the chromosome population during the process of evolution. The SAM determines the spectral similarity between two spectra (*i.e.*, target and reference) by calculating the angle between them in an n-dimensional space. To calculate the fitness function, half of the spectra of each species (17 spectra per species) were used for training purposes, and the remaining half for validation purposes. For each species, the average spectrum of training dataset was used as a reference spectrum.

#### 2.3.3. Selection

On the basis of fitness value (*i.e.*, the classification accuracy resulted from the SAM), the parent chromosomes were selected to reproduce offspring using random (roulette wheel) selection. The chromosomes with higher fitness values have a higher chance of being selected for reproduction and to generate a new chromosome.

#### 2.3.4. Preliminary Parameters and Chromosome Size

The initial parameters were configured as follows: Population size = 1,000, maximum number of generations = 500, crossover probability = 1, probability of mutation = 0.01, elite count (*i.e.*, the number of chromosomes with best fitness values in the current generation that are guaranteed to survive into the next generation; these chromosomes are called elite children) = 2.

**Figure 2.**
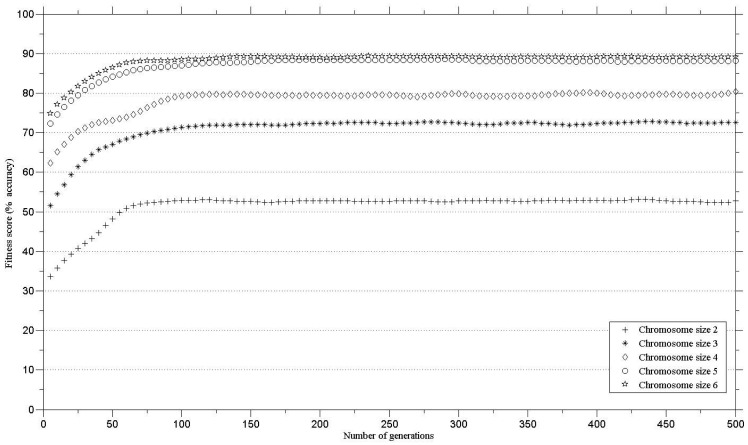
Performance of different sized chromosomes (number of bands in the chromosome) for the classification of 13 vegetation species.

In order to define the number of genes in a chromosome for maintaining high classification accuracy, the genetic algorithms were run with different gene numbers per chromosome. The minimum threshold for class separability (*i.e.*, classification accuracy) was set to 85% [25]. The minimum number of genes in a chromosome that exceeded the defined threshold was five. There was little increase in the classification accuracy when the genetic algorithm was executed with chromosomes with six bands (Figure 2). Therefore, a chromosome with five bands was chosen for further analysis.

The consistency of the genetic algorithms for discriminating vegetation species was checked by repeating the analysis 40 times. The data was reshuffled at the beginning of each run. The algorithms start with a random initial population and undergo selection (based on fitness score), crossover, mutation and elite count processes.

### 2.4. Evaluating the Performance of the Genetic Algorithm

The performance of the genetic algorithms in separating the species was assessed by using the Jeffries Matusita (J-M) distance [34]. The J-M distance is the average distance between two class density functions. The J-M distance takes into account the distance between class mean and the distribution of values from the means. Another advantage is that it can be executed over a number of bands (unlike M-statistics). The J-M distance is a parametric test, of which values range between 0 and 2, providing an easy comparison of class separability [1,3]. The J-M distance was calculated between each pair of species using the genetic algorithm based winner chromosome (using the bands selected on the basis of the genetic algorithm) as well as a randomly selected chromosome. Prior to conducting the tests, the distribution of the spectral emissivity values across selected waveband was tested for normality and the homogeneity of variance (homoscedasticity) was verified for every spectral band.

The average J-M distance between each species pair selected using the genetic algorithm's selected bands were compared with the average J-M distance derived from the randomly selected bands. The significance of difference in the J-M distances between the genetic algorithm based bands and randomly selected bands was tested using a t-test.

## 3. Results

### 3.1. Length of the Chromosome

The results (Figure 2) compare the fitness score against chromosome size for the thirteen species. The minimum number of genes in a chromosome that exceeded the defined threshold (classification accuracy of 85%) was five. There was no substantial increase in the classification accuracy using a six, compared to a five, band chromosome (Figure 2).

### 3.2. Band Pruning Based on Genetic Search Algorithms

**Figure 3.**
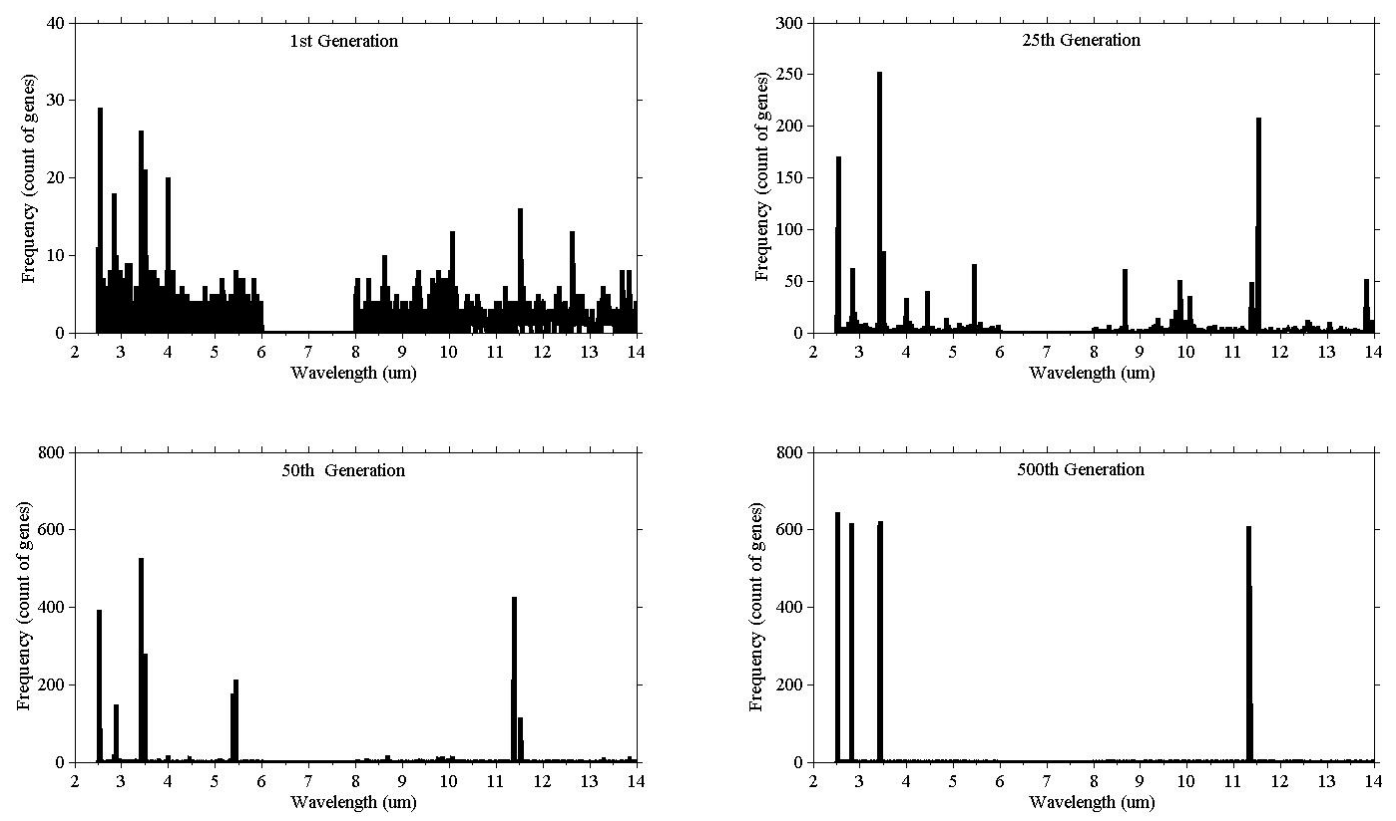
The graphical representation of gene convergence, the frequency (count of genes selected in the population) clustered around certain wavebands as the number of generations increases.

Illustrating the process of evolution, Figure 3 shows the result of a single run. The vertical (y) axis represents the count of the genes selected, while the horizontal axis (x) represents the wavelength. At the beginning (1st generation) the population consisted of randomly selected genes from all wavebands, and as the evolution proceeded the bands started to converge.

The overall classification accuracy using the winning chromosome genes are illustrated in Table 2. The results (Table 2) show that the classification accuracies of the winning chromosome were above the set threshold (85%) for both training and testing datasets.

**Figure 4.**
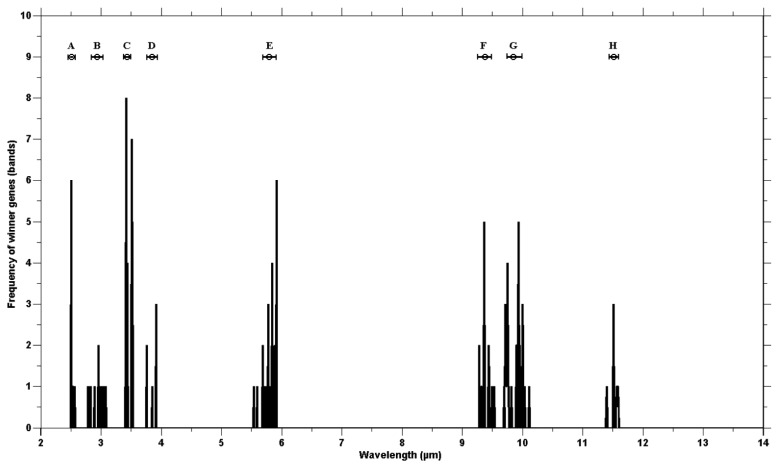
The vertical bars represent the number of winning genes at a certain wavelength region for all 40 runs. The horizontal bar at the top shows the spread (mean and standard deviation) of the spectral regions from which the winning bands are selected.

The genetic algorithm was run 40 times to check consistency. The wining chromosomes along with classification accuracies (based on the SAM) are reported in Appendix 1. The fitness scores of all winning chromosomes were above the defined threshold (classification accuracy over 85%). The frequency of the selected genes showed genes clustering around certain wavebands (Figure 4). The high frequency occurring at certain wavebands represents that waveband's importance for the separating of species. The selected genes were grouped into eight waveband regions based on the mean and standard deviation (Table 3). Five of those lie in the mid infrared (2.5–6 μm) and the remaining three regions belong to the thermal infrared (8–12 μm).

The eight waveband regions (where selected genes were grouped) correspond to the spectral wavebands positions of the Mid-wave infrared Airborne Spectrographic Imager (MASI600) and the Thermal infrared Airborne Spectrographic Imager (TASI600). The MASI600 and TASI600 are pushbroom hyperspectral sensors operating in the mid-wave infrared (3–5 μm) and thermal infrared (8–11.5 μm), having 64 continuous spectral bands. These sensors can acquire data at a maximum altitude of 3,048 m (above sea level). The spatial resolution varies between 1 m and 3.5 m (depending on the altitude of the platform) with a spatial coverage of 600 pixels. The first four waveband regions (B, C, D and E) correspond to the wavebands of MASI600 and the last three regions (F, G and H) lay within the spectral range of TASI600.

### 3.3. Evaluation of the Performance of Genetic Algorithm

The Jeffries Matusita (J-M) distances between different species pairs calculated using the bands selected by the genetic algorithm, were compared with the randomly selected bands. The five selected bands (resulting from the genetic algorithms and the random selection) were used to calculate the J-M distance between each species. The average J-M distance values of genetic algorithm based selected bands were higher than the value of randomly selected bands. The result of the t-test (Table 4) confirms that the differences between most J-M distances (74 out of 78 ≈95%), based on genetic algorithms and random selection, are statistically significant at a 95% confidence level (*p* ≤ 0.05).

The classification accuracy based on genetic algorithms selected bands was higher than results obtained by Ullah *et al.* [29]. They used One-way analysis of variance (ANOVA) coupled with a *post-hoc* Tuckey HSD test. The spectral features (bands resulting in the highest number of statistically significantly different pairs) were then manually selected. In this study, the genetic algorithms selected the bands, further improving the classification accuracy.

## 4. Discussion

This study tested the applicability of genetic algorithms for the selection of bands from the mid and thermal infrared emissivity spectra to discern thirteen vegetation species. The visible to shortwave infrared domain have been widely used for discriminating vegetation species, but mid to thermal infrared emissivity spectra have received little attention. The outcome of the study (Table 2 and Appendix 1) demonstrated that the genetic algorithm based selected bands (subset of five bands) achieved an overall accuracy of more than 85%.

The improved classification accuracy of the bands selected by genetic algorithms compared to the randomly selected bands could be attributed to the fact that genetic algorithms provide several possible solutions, evaluate them on the basis of an objective function and pick the best one for the next generation.

The validity of the combination of genetic algorithm based selected bands used for the spectral discrimination of vegetation species in the mid to thermal infrared emissivity spectra may be attributed to the spectral positioning of the selected bands. The emissivity spectra of the different plant species contain unique features due to the variation in physio-chemical composition of the superficial epidermal layer of the plant leaves. The emissivity signature of plant leaves is dominated by a feature associated with major classes of cellulous of the epidermis [26–28,35–38]. The selected waveband positions, between 2.5 to 6 μm, may be attributed to the physical makeup of the surface, as well as the water and chemical content of different plant leaves [27,39,40]. The clustering of the winning genes at around 3.00 μm may be due to OH band stretching and bending in the water molecule [26,27,40]. The selection of bands at the wavelength position of 3.44 μm may be due to the presence of different amounts of nonacosane (a compound in wax occurring on the leaf surface), as a result of the stretching of the CH_2_ bond of methylene in leaf surface waxes [41–43]. The stretching of carbonyl group (C=O) in ester has been linked to a spectral features at 5.80 μm [43,44]. Different amounts of leaf cutin and cutan (which are composed of esterified monomers) may be linked to the selection by the genetic algorithm of features at 5.80–5.92 μm (Figure 4). The bands selected between 9.40–9.70 μm (Figure 3) could be attributed to cellulose thickness, creating two prominent features at 9.47 μm and 9.68 μm, associated with the C-O band stretching [26,41]. The next spectral region winner bands were selected from (mean at 9.87 μm and standard deviation ±0.121 μm) may have resulted from differences in hemicellulose and other pectins [45,46]. The winning gene clustering at 11.50 μm (mean 11.50 and standard deviation ±0.121 μm, Figure 4) may have resulted from the presence of different aromatic compounds in the plant species [27].

Discriminating vegetation species using laboratory measured emissivity spectra is prerequisite for the future vegetation mapping campaigns from air-borne and space-borne data. However, there are a number of problems associated with extending this work to field level. The calibration of remotely sensed signals in the MIR (around 3 μm) is complicated by the difficulty associated with the overlap of reflected and emitted energy in the MIR. Other problems associated with field condition are the distance between target and sensor, spectral and spatial resolution, atmospheric condition, and seasonal changes. The cavity effect of plant leaves causes blackbody emittance in the TIR and reduces spectral contrast in the signal. The cavity effect problem is noticeable in small and needle leaved species and also in species with funnel-like leaf arrangements [28]. One could extend this study to a field, air-borne, and space-borne by using a sensing system with high signal to noise ratio (SNR) that allows small spectral differences in plant to be characterized.

## 5. Conclusions

This study has demonstrated the potential of genetic algorithms as band selectors using high resolution mid to thermal infrared emissivity spectra to differentiate between vegetation species at laboratory level. It is concluded that the bands selected by genetic algorithms are more useful for discriminating vegetation species than randomly selected bands are, when using laboratory emissivity spectra. The genetic algorithm based selected bands were actually found to have potential for floristic mapping. Bands selected with genetic algorithms may correspond to physiochemical characteristics of vegetation leaves (as seen in the previous studies) as leaves of different species possess unique surface materials. The genetic algorithm based selected bands help to understand the section of the electromagnetic spectrum that has a high potential for discriminating vegetation types, which may be useful when designing new sensors for vegetation studies. The outcome of this study is that the genetic algorithm band selection procedure can differentiate between plant species using laboratory measured thermal emission spectra. It would be very interesting to extend this work to the field and at airborne level with the advancement of hyperspectral thermal infrared sensors.

## Figures and Tables

**Table 1.** The plant species used for spectral measurements. Thirty five (35) leaves were measured per species.

**Species**	**Species code**

*Acer platanoides*	AP
*Asplenium nidus*	AN
*Cornus sericea*	CS
*Fallopia japonica*	FJ
*Ginkgo biloba*	GB
*Hedera helix*	HH
*Ilex opaca*	IL
*Liquidambar styraciflua*	LS
*Platanus orientalis*	PO
*Prunus laurocerasus*	PL
*Rhododendron caucasicum*	RH
*Spathiphyllum cochlearispathum*	SP
*Tilia platyphyllos*	TP

**Table 2.** The average confusion matrix (of 40 runs) for the training and testing dataset, the bands selected by genetic algorithms during training are used for evaluation by the testing dataset.

	PL	RH	SP	TP	AP	AN	CS	FJ	GB	HH	IL	LS	PO
PL	17	0	0	0	0	0	0	0	0	0	0	0	0
RH	0	15	0	0	0	0	0	1	0	0	0	1	0
SP	0	0	15	0	0	0	0	0	0	0	2	0	0
TP	0	0	0	17	0	0	0	0	0	0	0	0	0
AP	0	0	0	0	17	0	0	0	0	0	0	0	0
AN	0	0	0	0	0	17	0	0	0	0	0	0	0
CS	0	0	0	0	0	2	15	0	0	0	0	0	0
FJ	0	0	0	0	0	0	0	17	0	0	0	0	0
GB	0	0	0	0	0	0	0	0	17	0	0	0	0
HH	0	0	0	0	0	0	0	0	0	17	0	0	0
IL	0	0	0	0	0	0	0	0	0	0	17	0	0
LS	0	0	0	0	0	0	0	0	0	1	0	16	0
PO	0	0	0	0	0	0	0	0	0	0	0	0	17
Overall classification accuracy of training dataset = 96.83%													
	PL	RH	SP	TP	AP	AN	CS	FJ	GB	HH	IL	LS	PO
PL	12	0	0	0	0	0	0	0	0	0	2	3	0
RH	0	13	0	4	0	0	0	0	0	0	0	0	0
SP	0	0	12	0	0	0	5	0	0	0	0	0	0
TP	0	2	0	12	0	0	0	0	0	0	0	3	0
AP	0	0	0	0	17	0	0	0	0	0	0	0	0
AN	0	0	0	0	0	17	0	0	0	0	0	0	0
CS	0	0	0	0	0	0	17	0	0	0	0	0	0
FJ	0	0	0	0	0	0	0	17	0	0	0	0	0
GB	0	0	0	1	0	0	0	0	16	0	0	0	0
HH	0	0	0	0	0	0	0	0	0	17	0	0	0
IL	0	0	0	0	0	0	0	0	0	0	17	0	0
LS	0	0	0	0	0	0	0	0	0	0	0	17	0
PO	0	0	0	0	0	0	0	0	0	0	0	0	17
Overall classification accuracy of testing data = 90.50%													

**Table 3.** Summary of the clustering of selected genes (wavebands), the number of genes, spectral range, means wavelength location and standard deviation.

**Group**	**Spectral region**	**No. of genes**	**Wavelength range** **(μm)**	**Mean wavelength** **(μm)**	**Standard deviation** **(μm)**

A	Mid infrared	26	2.50–2.54	2.52	±0.020
B	Mid infrared	12	2.84–3.03	2.94	±0.097
C	Mid infrared	69	3. 40–3.48	3.44	±0.041
D	Mid infrared	6	3.77–3.93	3.85	±0.078
E	Mid infrared	30	5.70–5.90	5.80	±0.099
F	Thermal infrared	16	9.27–9.48	9.36	±0.107
G	Thermal infrared	35	9.74–10.00	9.87	±0.121
H	Thermal infrared	7	11.46–11.58	11.52	±0.064

**Table 4.** The results of t-test (*p*-values) between Jeffries Matusita (J-M) distances, calculated from genetic algorithms and randomly selected wavebands.

	AP	TP	AN	CS	FJ	GB	HH	IL	LS	PL	PO	RH	Sp
AP	-	0.00	0.02	0.11	0.01	0.00	0.00	0.00	0.01	0.13	0.02	0.01	0.01
TP	-	-	0.00	0.00	0.00	0.00	0.00	0.00	0.16	0.00	0.00	0.00	0.00
AN	-	-	-	0.01	0.00	0.00	0.00	0.00	0.00	0.00	0.00	0.00	0.00
CS	-	-	-	-	0.00	0.00	0.00	0.00	0.14	0.00	0.00	0.00	0.02
FJ	-	-	-	-	-	0.01	0.00	0.00	0.01	0.00	0.01	0.00	0.00
GB	-	-	-	-	-	-	0.03	0.00	0.00	0.01	0.00	0.00	0.03
HH	-	-	-	-	-	-	-	0.01	0.00	0.00	0.00	0.00	0.01
IL	-	-	-	-	-	-	-	-	0.00	0.00	0.00	0.00	0.00
LS	-	-	-	-	-	-	-	-	-	0.01	0.00	0.00	0.02
PL	-	-	-	-	-	-	-	-	-	-	0.00	0.00	0.00
PO	-	-	-	-	-	-	-	-	-	-	-	0.01	0.00
RH	-	-	-	-	-	-	-	-	-	-	-	-	0.00
Sp	-	-	-	-	-	-	-	-	-	-	-	-	-

## Appendix

**Appendix 1.** The winning genes at each run and their fitness score.

**Number of runs**	**The winning genes (bands in μm)**					**Fitness score (% Accuracy)**
1	2.567	3.420	3.511	5.797	9.973	88.55
2	3.417	3.511	3.917	9.711	11.511	90.58
3	2.540	3.023	3.415	9.720	11.563	90.50
4	2.528	3.416	3.425	5.913	11.501	90.14
5	3.417	3.511	3.917	9.711	11.511	90.58
6	2.503	2.984	3.413	9.748	11.588	87.15
7	3.421	3.523	5.913	9.361	9.934	87.33
8	3.422	3.524	5.833	9.361	9.897	85.97
9	2.530	2.973	3.414	9.533	11.575	89.69
10	2.504	2.974	3.418	9.058	9.925	91.12
11	2.534	3.413	3.509	5.833	9.739	89.24
12	3.420	3.511	3.758	5.893	9.954	89.69
13	2.540	2.801	3.415	5.537	9.437	89.50
14	2.536	3.082	3.412	9.319	9.897	85.60
15	2.505	3.417	5.265	9.285	9.729	89.43
16	2.505	3.418	3.427	3.853	11.397	89.24
17	2.503	3.418	5.822	9.285	9.489	86.55
18	2.503	2.786	3.417	5.775	9.446	93.76
19	2.503	2.957	3.418	9.319	9.748	86.20
20	3.420	3.424	5.737	5.846	10.011	87.72
21	3.417	3.511	3.917	9.711	11.511	90.58
22	2.503	2.890	3.418	5.781	9.812	86.64
23	2.528	3.418	3.422	5.740	10.011	90.13
24	2.503	3.416	5.591	5.913	9.720	91.38
25	2.562	3.412	5.692	9.437	10.109	91.71
26	3.423	3.515	5.724	5.916	9.925	88.28
27	3.058	3.422	5.804	5.913	9.748	86.34
28	2.553	3.412	5.775	5.775	9.934	87.83
29	2.536	3.416	5.791	9.335	9.693	90.95
30	3.421	3.523	5.913	9.361	9.934	87.33
31	3.411	3.437	5.686	5.913	10.002	86.47
32	3.421	3.523	5.913	9.361	9.934	87.33
33	2.503	3.418	3.437	5.846	9.906	89.00
34	2.511	3.408	3.443	5.846	10.002	85.66
35	3.407	3.425	3.523	9.766	11.520	92.53
36	2.535	2.832	3.420	9.310	10.040	87.33
37	3.421	3.523	5.913	9.361	9.934	87.33
38	2.518	3.417	3.511	5.788	9.757	92.77
39	2.503	3.407	3.437	5.846	9.906	89.00
40	3.411	3.437	5.686	5.913	10.002	86.47
